# Aortic arch anomaly in an adult patient: a case of right aortic arch with aberrant left subclavian artery and Kommerell's diverticulum

**DOI:** 10.1590/0100-3984.2015.0087

**Published:** 2016

**Authors:** Alexandre Ferreira Silva, José Antônio dos Santos

**Affiliations:** 1 Ecotomo S/C Ltda., Belém, PA, Brazil.; 2 Dimagem - Diagnóstico por Imagem, Belém, PA, Brazil.

**Figure 1:**
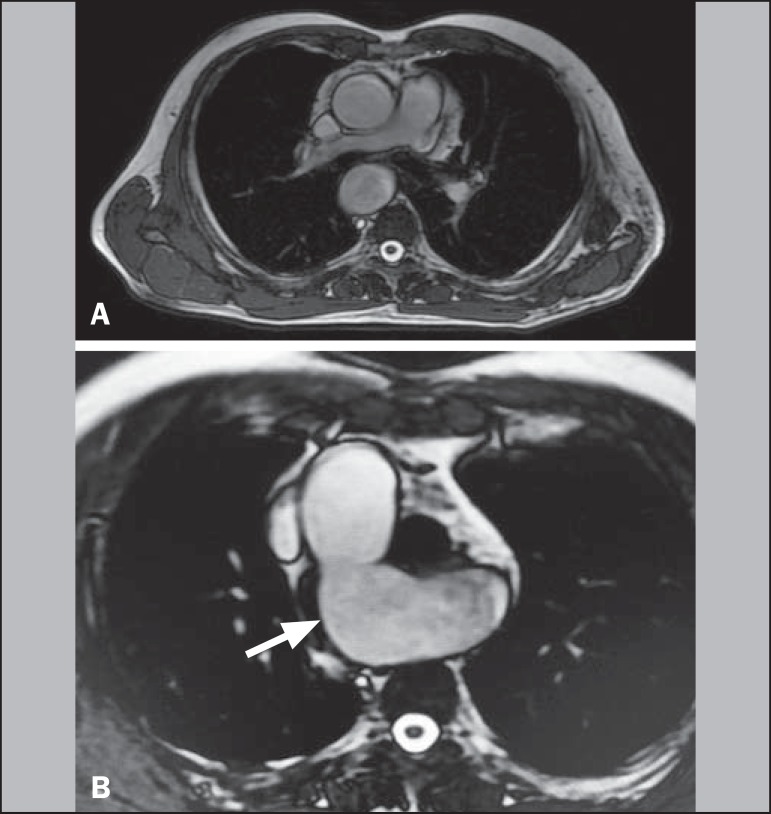
**A,B:** Axial T2-weighted spin-echo magnetic resonance imaging showing right aortic arch (arrow).

**Figure 2:**
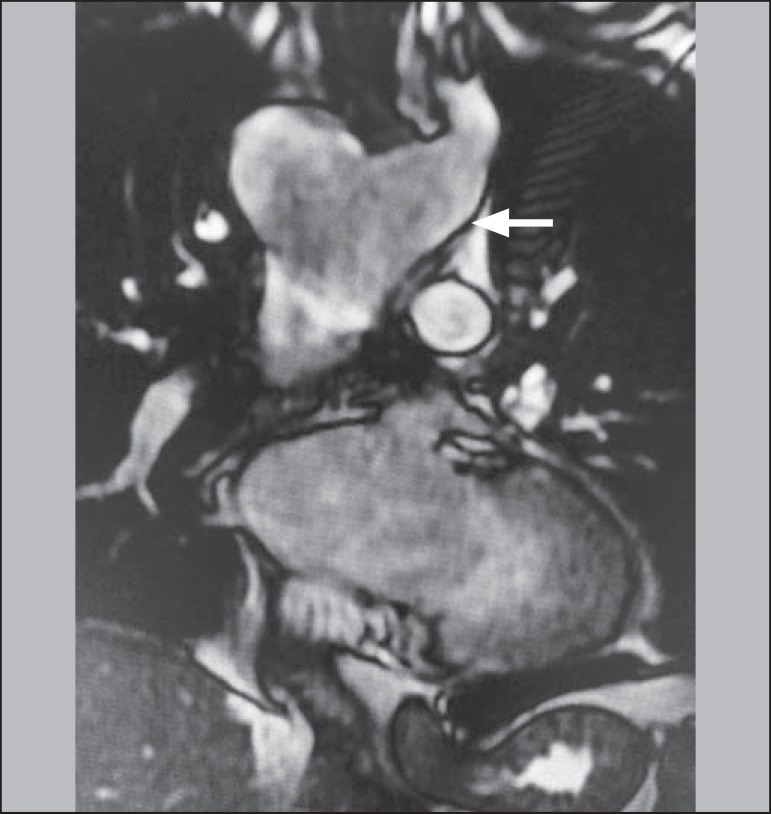
Coronal T2-weighted spin-echo magnetic resonance imaging showing Kommerell's diverticulum (arrow).

*Dear Editor*,

We report the case of a 54-year-old male presenting with vague symptoms of discomfort when swallowing. The patient underwent magnetic resonance imaging of the chest. The examination showed right aortic arch with an aberrant left subclavian artery and Kommerell's diverticulum (Figures 1 and 2).

Thoracic diseases of vascular origin have been the subject of a number of recent publications in the radiology literature of Brazil^(1-5)^. First described by Fioratti et al., right aortic arch is an uncommon birth defect, of unknown cause, occurring in 0.05% of the general population. It is often asymptomatic but can be accompanied by dysphagia and complications arising from the formation of an aneurysm. Such an aneurysm generally occurs at the origin of the left subclavian artery and is known as Kommerell's aneurysm or Kommerell's diverticulum, which can cause compression of mediastinal structures or can rupture spontaneously^(6-13)^. In children, the symptoms can also be associated with existing congenital cardiac abnormalities^(7)^.

Various systems for classifying right aortic arch have been proposed. The most widely used classification system is that devised by Edwards, who described three main types of right aortic arch: type I, with mirror-image branching of the major arteries; type II, with an aberrant subclavian artery; and type III, with an isolated subclavian artery (connected to the pulmonary artery via the ductus arteriosus)^(8)^. In the case presented here, the variant was classified as an Edwards type II right aortic arch, which accounts for approximately 40% of all cases^(7)^.

In an autopsy study cited by Faucz et al.^(7)^, 50% of cases of right aortic arch were associated with an aberrant left subclavian artery, which can be located behind the esophagus (in 80%), between the trachea and the esophagus (in 15%), or anterior to the trachea (in 5%). In some cases, right aortic arch is associated with a congenital heart defect^(7,9,10)^.

The treatment of right aortic arch is generally surgical and is quite complex. Preoperative imaging tests are extremely important for the surgical planning, which relies heavily on knowledge of the anatomical distribution of the local structures, as well as of the size and extent of the aneurysm. Although outpatient treatment is an option, endovascular repair has been performed successfully^(7,11)^.

The indication for surgical intervention in right aortic arch continues to be a subject of debate. Surgical intervention is considered an acceptable option when the diameter of the orifice of the diverticulum is > 30 mm or the diameter of the descending aorta adjacent to the diverticulum is > 50 mm^(11)^.
